# Cytotoxic Compounds from *Aloe megalacantha*

**DOI:** 10.3390/molecules22071136

**Published:** 2017-07-07

**Authors:** Negera Abdissa, Sebastian Gohlke, Marcel Frese, Norbert Sewald

**Affiliations:** 1 Department of Chemistry, Organic and Bioorganic Chemistry, Bielefeld University, P.O. Box 100131, 33501 Bielefeld, Germany; sebastian.gohlke@uni-bielefeld.de (S.G.); marcel.frese@uni-bielefeld.de (M.F.); 2 Department of Chemistry, College of Natural Sciences, Jimma University, P.O. Box 378, 251 Jimma, Ethiopia

**Keywords:** Asphodelaceae, *Aloe megalacantha*, roots, quinone, anthraquinone, cytotoxicity

## Abstract

Phytochemical investigation of the ethyl acetate extract of the roots of *Aloe megalacantha* led to the isolation of four new natural products—1,8-dimethoxynepodinol (**1**), aloesaponarin III (**2**), 10-*O*-methylchrysalodin (**3**) and methyl-26-*O*-feruloyl-oxyhexacosanate (**4**)—along with ten known compounds. All purified metabolites were characterized by NMR, mass spectrometric analyses and comparison with literature data. The isolates were evaluated for their cytotoxic activity against a human cervix carcinoma cell line KB-3-1 and some of them exhibited good activity, with aloesaponarin II (IC_50_ = 0.98 µM) being the most active compound.

## 1. Introduction

*Aloe* is the largest genus in the Asphodelaceae family and comprised of more than 400 species [1], ranging from diminutive shrubs to large trees distributed across Africa, with the major diversity in South Africa [2]. *Aloe* is represented in East Africa by 83 species, of which 38 grow naturally in Ethiopia, including 15 endemic species [2,3,4]. It has long medicinal and economic history since 1500 BC [5]. The *Aloe* gel found in the interior of the leaves has been extensively used for the treatment of human and livestock diseases [6,7]. In fact, *Aloe vera* is well known as a commercial crop in many countries for its extensive use in cosmetic and pharmaceutical industries [6]. The plants produce different varieties of polyketides; mainly anthraquinones have been reported to possess antimicrobial [8], antimalarial [9], and cytotoxic [10] activities.

*Aloe megalacantha* is one of the species growing in Ethiopia and has been commonly used by traditional healers in the Western and Southern part of Ethiopia for the treatment of wounds. While the genus *Aloe* is the most extensively investigated species due to its rich medicinal value and chemotaxonomic aspect, no phytochemical analysis and biological activities of *A. megalacantha* have been reported. Thus, as part of our ongoing search for new bioactive natural products of plants from the Asphodelaceae family [11,12,13], we report the isolation of four new natural products **1**–**4**, along with ten known compounds (one coumarin and nine anthraquinones). The cytotoxicity of the compounds against the human cervix carcinoma cell line KB-3-1 is also reported.

## 2. Results and Discussion

**Figure 1:**
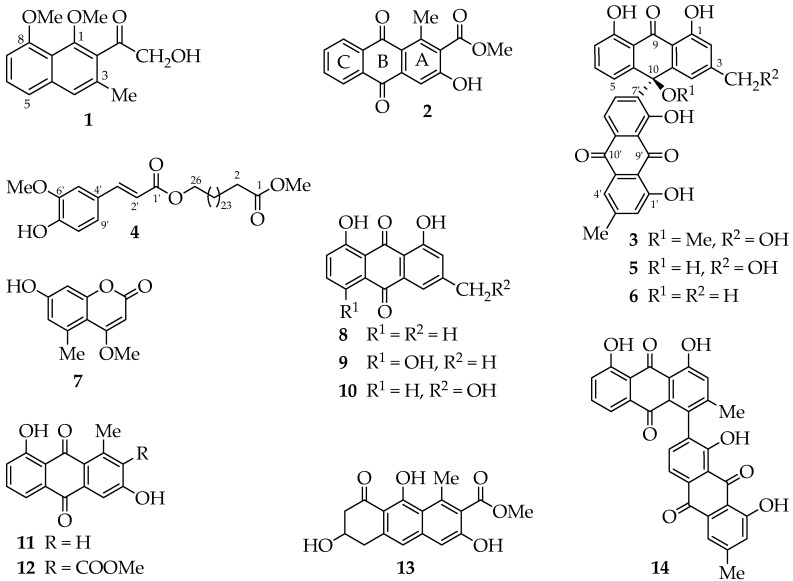
Structures of the compounds.

Chromatographic separation of the ethyl acetate extract of *A. megalacantha* roots furnished fourteen compounds (Figure 1). All spectroscopic data, including UV, IR and NMR data, were in good agreement with the reported data for the known compounds chrysalodin (**5**) [14], 10-(chrysophanol-7′-yl)-10-hydroxychrysophanol-9-anthrone(**6**) [9,15], 7-hydroxy-4-methoxy-5-methylcoumarin (**7**) [16], chrysophanol (**8**) [17], helminthosporin (**9**) [18], aloeemodin (**10**) [19], aloesaponarin II (**11**) [20], aloesaponarin I (**12**) [21], aloesaponol I (**13**) [20,21] and asphodelin (**14**) [22]. It is worth to point out that this is the first report of 7-hydroxy-4-methoxy-5-methylcoumarin and its kind from the genus and most probably from the family of Asphodelaceae, having been previously reported from stem bark of *Toona ciliate* [16].

Compound **1** was isolated as colourless amorphous solid and the molecular formula C_15_H_16_O_4_, deduced from its HR-ESI-MS ([M + Na]^+^ *m*/*z* 283.0916), indicated eight degrees of unsaturation. The UV (λ_max_ 252, 276 nm) and IR (ν_max_ 1701, 1618, 1571, 1346 cm^−1^) spectra revealed absorptions for conjugated ketone and aromatic moieties. The ^13^C-NMR spectrum (Table 1) revealed ten *sp*^2^ hybridized carbon atoms of a naphthalene skeleton. The presence of a hydroxylmethylketone (δ_H_ 4.55, δ_C_ 70.5; δ_C_ 207.5), a methyl (δ_H_ 2.30, δ_C_ 19.0) and two methoxy (δ_H_ 3.73, δ_C_ 64.1; δ_H_ 3.99, δ_C_ 56.3) substituents were also evident. The ^1^H-NMR spectrum (Table 1) showed three mutually *ortho*/*meta*-coupled aromatic protons at δ_H_ 6.97 (dd, *J =* 7.6, 1.2 Hz, 1H), 7.44 (t, *J =* 7.8 Hz, 1H) and 7.40 (dd, *J =* 7.8, 1.4 Hz, 1H), corresponding to H-5, H-6, and H-7, respectively.

**Figure 2:**
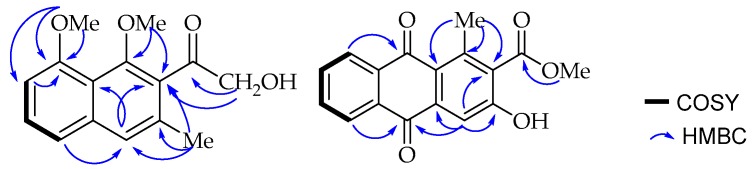
COSY (bold lines) and key HMBC (blue arrows) correlations of **1** and **2**

One of the methoxy groups (δ_H_ 3.99, δ_C_ 56.3) is present at C-8 (δ_C_ 157.0) according to its long range HMBC correlation (Figure 2) with the neighbouring carbons, C-7 (δ_C_ 106.7) and C-8 (δ_C_ 157.0). A broad singlet aromatic proton at δ_H_ 7.48 (H-4) showed a long-range NOE interaction in the NOESY spectrum with H-5 (δ_H_ 6.97) and the methyl group (δ_H_ 2.30; δ_C_ 19.0) located as expected according to biosynthetic considerations at C-3 (δ_C_ 133.6). The substituents at C-1 (δ_C_ 154.8) and C-2 (δ_C_ 132.0) were, therefore, established to be the methoxy (δ_H_ 3.73, δ_C_ 64.1) and the hydroxymethylketone unit (δ_H_ 4.55, δ_C_ 70.5; δ_C_ 207.5) respectively, based on a *^3^J*_C,H_ HMBC correlation of both H-4 (δ_H_ 7.48) and the hydroxymethyl protons (δ_H_ 4.55) with C-2 (δ_C_ 132.0). These placements were further confirmed by the long range HMBC correlations observed between methoxy protons (δ_H_ 3.73) and its nearby C-1 and C-2 carbons. Interestingly, the down-field shifted signal at δ_C_ 207.5 for the aryl ketone in the ^13^C-NMR spectrum is consistent with a group being *ortho*-disubstituted, resulting in a deviation from planarity as a result of steric repulsion. Therefore, based on the above spectroscopic evidence, the new compound was characterized as 2-hydroxymethylacetyl-1,8-dimethoxy-3-methylnaphthalene, which was given the trivial name 1,8-dimethoxynepodinol (**1**).

Compound **2** was isolated as yellow solid. The HR-ESI-MS provided a pseudomolecular ion peak at *m*/*z* 297.0753 [M + H]^+^, corresponding to the molecular formula of C_17_H_12_O_5_. The UV spectrum (λ_max_ 264, 298, 336, 371 nm) and NMR spectra suggested an anthraquinone skeleton [23]. The ^13^C-NMR spectrum in total contains seventeen carbon signals (Table 1), among them three carbonyl signals (δ_C_ 184.0, 183.5 and 168.3). The former two signals were attributed to a quinone system and the latter to the carboxylic acid methyl ester. In the ^1^H-NMR spectrum (Table 1), four mutually coupled aromatic protons of AA′BB′ spin pattern centered at δ_H_ 8.19 (dd, *J* = 7.7, 1.4 Hz, 1H), 7.86 (td, *J* = 7.5, 1.4 Hz, 1H), 7.91 (td, *J* = 7.5, 1.5 Hz, 1H) and 8.24 (dd, *J* = 7.7, 1.5 Hz, 1H) were assigned to H-5, H-6, H-7, and H-8, respectively, located at the disubstituted ring C. A singlet at δ_H_ 7.75 was assigned to H-4 in ring A, which otherwise is fully substituted with a methyl group shifted downfield to *δ*_H_ 2.71 due to the deshielding effect of the neighbouring carbonyl group attached to C-1 (δ_C_ 142.3). Furthermore, a carboxylic acid methyl ester (δ_H_ 3.95; δ_C_ 52.9, δ_C_ 168.3) at C-2 (δ_C_ 130.8) (based on biosynthetic considerations) and a hydroxy group at C-3 (δ_C_ 159.0). These assignments were in agreement with the biosynthetic consideration that appear to have been formed through folding of the octaketide chain in an unusual way as in aloesaponarin II [24] and further confirmed by the HMBC correlation (Figure 2) of methyl protons (δ_H_ 2.71) with C-1, C-1a (δ_C_ 125.3) and C-2; and H-4 (δ_H_ 7.75) with C-1a, C-2 and C-3.

The ^13^C-NMR spectrum further showed the presence of five *sp*^2^ methines (δ_C_ 112.8, 127.2, 128.0, 134.4, 135.5), one methyl (δ_C_ 20.0) and seven *sp*^2^ quaternary carbons (δ_C_ 125.3, 130.8, 133.5, 136.2, 138.3, 142.3, 159.0). These data are consistent with the compound being methyl 3-hydroxy- 1-methyl-9,10-dioxo-9,10-dihydroanthracene-2-carboxylate (Figure 1) which was given the trivial name aloesaponarin III. This is the first report on the natural occurrence of compound **2** that had previously been reported as a synthetic intermediate [25].

Compound **3** was isolated as yellow amorphous powder and was assigned the molecular formula C_31_H_22_O_9_ based on the HR-ESI-MS analysis (negative mode; [M − H]^−^ *m*/*z* 537.1863) and ^13^C-NMR data (Table 1). The positive mode of HR-MS did not show the molecular ion peak, instead the demethoxylated pseudo molecular ion peak [M – OCH_3_]^+^ (*m*/*z* = 507.1034) was observed similar to the related dimeric anthraquinones [9,12]. Its UV–vis spectrum showed characteristic absorptions (λ_max_ 215, 261, 384, 432 nm) consistent with an anthrone-anthraquinone dimer [14]. The presence of four highly downfield shifted proton signals (δ_H_ 12.36, 12.39, 12.10, 11.76) corresponding to hydroxyl groups involved in hydrogen bonding and three carbonyl groups (δ_C_ 193.1, 192.5, 182.0), a methyl (δ_H_ 2.43; δ_C_ 22.4) and hydroxymethyl (δ_H_ 4.64; δ_C_ 64.5) groups (Table 1) supported the suggestion that the compound is a dimer of 1,8-dihydroxyanthraquinone/anthrone derivative similar to chrysalodin (**5**) [14].

**Figure 3:**
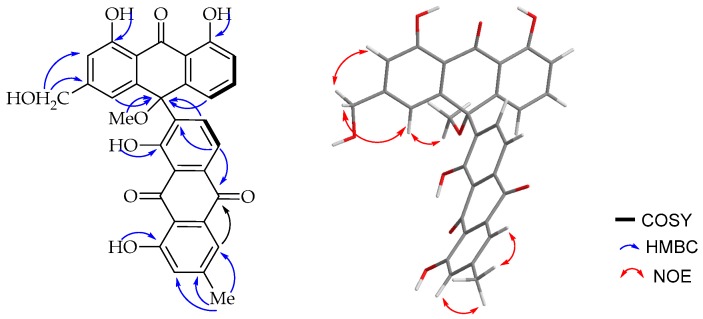
COSY (bold lines), key HMBC (blue arrows) and key NOE (red arrows) correlations of **3**.

The ^1^H-NMR spectroscopic (Table 1) features were virtually identical to those of chrysalodin (**5**) with one-half of the molecule constituting aloeemodinanthrone, consisting of three mutually *ortho*/*meta*-coupled aromatic protons resonating at δ_H_ 6.77 (dd, *J* = 8.0, 1.1Hz, 1H), 7.43 (t, *J* = 8.2 Hz, 1H) and 6.95 (dd, *J* = 8.5, 1.1 Hz, 1H) for H-5, H-6 and H-7 respectively, *meta*-coupled protons at δ_H_ 6.76 (d, *J* = 1.5 Hz, H-2) and 6.99 (d, *J* = 1.5 Hz, H-4), and broad singlet proton signal at δ_H_ 4.64 for a hydroxymethyl group. The other half of the molecule constitutes a chrysophanol moiety, where the presence of *meta*-coupled aromatic protons δ_H_ 7.60 (H-2′) and 7.02 (H-4′) with the biosynthetically expected methyl group (δ_H_ 2.43; δ_C_ 22.4) being located at C-3′ (δ_C_ 149.6) and a pair of downfield shifted *ortho*-coupled protons at δ_H_ 7.89 (d, *J* = 8.0 Hz, 1H) and δ_H_ 8.85 (d, *J* = 8.0 Hz, 1H) for H-5′ and H-6′, respectively, clearly indicated the attachment of the aloeemodin moiety at C-7′ (δ_C_ 141.7) as in chrysalodin [14]. The HMBC spectrum (Figure 3) showed a ^3^*J*_C,H_ correlation between H-6′ (δ_H_ 8.85) and C-10 (δ_C_ 75.6) supporting the suggestion that C-7′ is the point of attachment to the former half of the molecule. The only notable difference is the presence of a singlet integrating for three protons at δ_H_ 2.87 attached to the carbon resonating at δ_C_ 50.7 showing a HMBC correlation to C-10 (δ_C_ 75.6). The upfield chemical shift value of the methoxy group (δ_H_ 2.87) may be ascribed to anisotropy. It also showed an NOE (Figure 3) correlation with H-4. The optical activity (${\left[\alpha \right]}_{D}^{20}$ = +46 (*c* 0.5, CH_2_Cl_2_)) and the CD spectrum confirms the chirality of compound **3**. The absolute configuration at the chiral center was then established in comparison with a related dimeric anthraquinone, abiyquinone B [26]. Compound **3** has opposite optical activity and a strong negative Cotton effect at shorter wavelength close to 235 nm in the CD spectrum. Based on the above spectroscopic evidence, the third compound is (*S*)-10-(chrysophanol-7′-yl)-10-methoxy-aloeemodin-9-anthrone that was given the trivial name 10-*O*-methylchrysalodin (**3**).

Compound **4** was obtained as a colourless solid. The ESI-MS (*m*/*z* 625 (for [M + Na]^+^) and HR-MS (*m*/*z* 603.5395 (for [M + H]^+^) along with the NMR data was compatible with the molecular formula C_37_H_62_O_6_. It has a UV absorption at λ_max_ 213, 278 nm. Its IR spectrum indicated the presence of hydroxyl (3410 cm^−1^) and conjugated carbonyl (1637 cm^−1^) functionalities. The ^1^H-NMR spectrum (in CDCl_3_) exhibited signals corresponding to a ferulic acid moiety with a pair of doublets at δ_H_ 6.29 and 7.61 with a vicinal coupling constant *^3^J*_HH_ = 15.9 Hz. This indicated a *trans* configuration and the signals were assigned to H-2′ and H-3′, respectively. These protons showed HMBC correlations with the ester carbonyl carbon at δ_C_ 167.5, suggesting that these protons were part of an α,β-unsaturated ester moiety. The three aromatic proton signals at δ_H_ 7.03 (*J*_HH_ = 1.9 Hz), 6.92 (*J*_HH_ = 8.2 Hz) and 7.07 (*J*_HH_ = 8.2, 1.9 Hz) were assigned to H-5′, H-8′, and H-9′, respectively based on their HH-COSY interactions and coupling constants. These assignments were further supported by the HMBC correlations of the downfield shifted proton H-3′ (δ_H_ 7.61) with C-5′ (δ_C_ 109.4) and C-9′ (δ_C_ 123.2) that in turn have HMQC correlations with H-5′ and H-9′, respectively. The ^1^H-NMR spectrum further showed the presence of a singlet at δ_H_ 5.84 corresponding to a phenolic hydroxy group, two singlets integrating for three protons each at δ_H_ 3.66 and 3.93 for two methoxy groups, two triplets at δ_H_ 4.19 and 2.30 for methylene groups (attached to oxygen and carbonyl respectively, deduced from their chemical shift) and multiplets corresponding to long methylene chain. The ^13^C-NMR spectrum contains signals corresponding to two ester carbonyl carbons (δ_C_ 174.5, 167.5), five aromatic/olefinic methine groups (δ_C_ 144.8, 123.2, 115.8, 114.8, 109.4), two oxygenated aromatic quaternary carbons (δ_C_ 148.0, 146.9), one oxymethylene (δ_C_ 64.8), two methoxy (δ_C_ 56.1, 51.6) and a number of methylene (34.3–24.9) carbons. The position of one methoxy group (δ_H_ 3.93; δ_C_ 56.1) was established at C-6′ (δ_C_ 148.0) according to its HMBC correlation to C-6′, while the hydroxy group at δ_H_ 5.84 showed a correlation to C-7′ (δ_C_ 146.9). The other methoxy group (δ_H_ 3.66; δ_C_ 51.6) corresponds to a methyl ester deduced from its upfield shifted (δ_C_ 51.6) signal [12] and from its HMBC correlation with the ester carbonyl (δ_C_ 174.5). Comparison of these spectroscopic data with the literature revealed that the compound was very similar to ω-feruloyloxyacid [27] and ethyl 24-(feruloyloxy)docosanoate [28]. The only difference was the absence of a methoxy group and the length of the aliphatic chain. The NMR spectra and ESI-MS (*m*/*z* 625 [M + Na]^+^, 601 [M−H]^−^) were in accordance with the presence of a ω-oxygenated C_26_ fatty acid chain, like ω-feruloyloxyacids with along aliphatic chain (C_19_–C_27_), reported from peat soil [27]. Based on the spectroscopic data and the literature information, the structure of compound **4** was concluded to be methyl (*E*)-26-((3-(4-hydroxy-3-methoxyphenyl)acryloyl)oxy)hexacosanoate that was given the trivial name methyl 26-*O*-feruloyl-oxyhexacosanoate.

The isolates were evaluated for their cytotoxic activities against the human cervix cancer cell line KB-3-1, with cryptophycin-52 (IC_50_ = 1.3 × 10^−5^ µM) and griseofulvin (IC_50_ = 19.0 µM) as positive controls, as described in previous reports [29]. Two compounds, aloesaponarin II (**11**) and aloesaponarin I (**12**) showed good cytotoxic activity with IC_50_ values of 0.98 µM and 16.00 µM, respectively whereas the dimeric anthraquinone asphodelin (**14**) showed weak activity (>60.00 µM). The other compounds showed little or no inhibitory activities. The activity of aloesaponarin II is sixteen times higher compared to aloesaponarin I; however, both possess similar quinone nuclei with a methyl and hydroxyl substitutions at similar position. The only difference is the presence of an electron withdrawing methyl ester group in aloesaponarin I, which may negatively influence the cytotoxic activity of the compound.

It is worth mentioning that several cancer chemotherapeutic agents such as doxorubicin, mitomycin C, and mitoxantrone contain a common structural quinone nucleus. This chemical structure allows them to be involved in multiple biological oxidative processes [30]. The reduction of the quinone leads to toxic species (semiquinone anion radical and hydroquinone) which act selectively in hypoxic tissues like tumor cells [31].

## 3. Materials and Methods

### 3.1. General Information

Melting points were recorded on B-540 melting point apparatus (Büchi, Flawil, Switzerland). Column chromatography was carried out on silica gel (0.06–0.2 mm, Merck, Darmstadt, Germany) deactivated with 3% aq. oxalic acid. Gel filtration was carried out on Sephadex LH-20 (GE Healthcare, Uppsala, Sweden). Analytical TLC was performed on Merck pre-coated silica gel 60 F_254_ plates (Merck, Darmstadt, Germany). UV spectra were recorded on a UV-3100PC spectrophotometer (VWR international, Darmstadt, Germany). High Resolution ESI-MS was done on a Micromass AC-TOF micro mass spectrometer (Micromass, Agilent Technologies 1200 series, Waldbronn, Germany). Optical rotations were measured on a P-1020 polarimeter (JASCO, Tokyo, Japan). CD spectra were measured on a JASCO J-810 CD spectrometer (JASCO, Tokyo, Japan). IR spectra were recorded on a Nicolet 380 FT-IR spectrometer (Thermo Electron Corporation, Madison, WI, USA). 1D NMR and 2D (COSY, HSQC, HMBC, NOESY) NMR spectra were recorded on an Avance 500 MHz spectrometer (Bruker, Rheinstetten, Germany) at 500 MHz (^1^H) and 125 MHz (^13^C) at 298 K using the residual solvent peaks as a reference.

### 3.2. Plant Materials

The roots of *A. megalacantha* were collected from West Arsinegele (7°22′26.2″ N 38°40′02.6″ E), Ethiopia, 240 Km away from Addis Ababa on the way to Shashemene in September 2016. The plant material was identified by professional botanist at Jimma University (Dr. Kitessa Hundera) and the voucher specimen (voucher number NA-07/16) has been deposited in the Jimma University Herbarium.

### 3.3. Extraction and Isolation

The air dried and powdered roots of *A. megalacantha* (840 g) were extracted exhaustively with ethyl acetate (3 L) four times each for 24 h at room temperature. The extract was then concentrated under reduced pressure using a rotary evaporator to yield 34 g of brown extract. A portion of the extract (30 g) was subjected to column chromatography on oxalic acid deactivated silica gel (400 g) and the column was eluted with petroleum ether containing increasing amounts of ethyl acetate to give 37 fractions each of ca. 250 mL.

Fractions 6–8 (eluting at 5% *v*/*v* ethyl acetate in petroleum ether) were further separated by repeated gel chromatography on Sephadex LH-20 (eluting with CH_2_Cl_2_/MeOH, 1:1) to give chrysophanol (4.0 mg), helminthosporin (3.0 mg) and compound **4** (2.1 mg). Fractions 15–18 (eluting at 10% *v*/*v* ethyl acetate in petroleum ether) were combined based on their TLC profile. Purification by column chromatography (column size: 60 cm length and 3 cm diameter) with an increasing gradient of ethyl acetate in petroleum ether gave asphodelin (8.0 mg), compound **2** (2.8 mg) and 10-(chrysophanol-7′-yl)-10-hydroxychrysophanol-9-anthrone (6.1 mg). Fractions 19–24 (eluting at 24% *v*/*v* ethyl acetate in petroleum ether) containing mixtures of five compounds were combined and subsequently subjected to column chromatography (column size: 80 cm length and 4 cm diameter) on silica gel (300 g) impregnated with oxalic acid (eluent: increasing gradient of ethyl acetate in petroleum ether). This was followed by further purification of the fractions on Sephadex LH-20 (dichloromethane/methanol, 1:1) yielding aloesaponarin I (4.8 mg), aloesaponarin II (3.9 mg), 4,7-dihydroxy-5-methylcoumarin (8.7 mg), compound **1** (4.2 mg) and aloeemodin (10.2 mg). Fractions 26–28 (eluting at 35% *v*/*v* ethyl acetate in petroleum ether) were collected as a yellow coloured solution. Further purification by gel chromatography using Sephadex LH 20 (eluting with CH_2_Cl_2_/MeOH; 1:1) gave compound **3** (3.4 mg) and chrysalodin (4.2 mg). Fractions 30–32 (eluting at 60% *v*/*v* ethyl acetate in petroleum ether) gave a colourless precipitate that was filtered and washed with dichloromethane/ethyl acetate mixture yielding aloesaponol I (21.0 mg).

*1,8-Dimethoxynepodinol* (**1**): Colourless amorphous solid. m.p. 132–134 °C. UV (CH_3_CN): λ_max_ (logε) = 252 (2.62), 276 (2.54) nm. IR (CH_2_Cl_2_) ν_max_ cm^−1^ 3472, 1701, 1618, 1571, 1346. ^1^H- and ^13^C-NMR (Table 1). ESI-MS (rel. int.): *m*/*z* = 543 (100, [2 M + Na]^+^), 283 (57, [M + Na]^+^). HR-ESI-MS *m*/*z* = 283.0916, [M + Na]^+^ (calculated for C_15_H_16_O_4_Na, 283.0946).

*Aloesaponarin III* (**2**): Yellow solid. m.p. 257–259 °C. UV (CH_3_CN): λ_max_ (logε) = 264 (2.80), 298 (2.73), 336 (2.63), 371 (2.44) nm. IR (CH_2_Cl_2_) ν_max_ cm^−1^ 3363, 2948, 1732, 1662, 1577, 1235, 712.^1^H- and ^13^C-NMR (Table 1). ESI-MS (rel. int.) *m*/*z* = 615 (21, [2 M + Na]^+^), 319 (43, [M + Na]^+^), 297 (100, [M + H]^+^). HR-ESI-MS *m*/*z* = 297.0753, [M + H]^+^(calculated for C_17_H_13_O_5_, 297.0762).

*10-O-Methylchrysalodin* (**3**): Yellow amorphous solid. m.p. 228–230 °C. UV (CH_2_Cl_2_): λ_max_ (logε) = 215 (3.18), 261 (3.14), 384 (2.94), 432 (2.66) nm. ${\left[\alpha \right]}_{D}^{20}$ +46° (c 0.5, CH_2_Cl_2_). ^1^H- and ^13^C-NMR (Table 1). ESI-MS (rel. int.) *m*/*z*= 507 (18, [M–OMe]^+^), 537 (12, [M − H]^−^). HR-ESI-MS *m*/*z* = 537.1863, [M − H]^−^ (calculated for C_31_H_21_O_9_, 537.1855).

*Methyl 26-O-feruloyl-oxyhexacosanoate* (**4**): Colourless solid. m.p. 187–189 °C. UV (CH_2_Cl_2_): λ_max_ (logε) = 213 (2.91), 278 (2.87) nm. IR (CH_2_Cl_2_) ν_max_ cm^−1^ 3410, 1762, 118, 1637. ^1^H-NMR (CDCl_3_) δ_H_ 7.61 (1H, *d*, *J* = 15.9 Hz, H-3′), 7.07 (1H, *dd*, *J* = 1.9, 8.2 Hz, H-9′), 7.03 (1H, *d*, *J* = 1.9 Hz, H-5′), 6.92 (1H, *d*, *J* = 8.2 Hz, H-8′), 6.29 (1H, *d*, *J* = 15.9 Hz, H-2′), 5.84 (1H, *s*, 7′-OH), 4.19 (2H, *t*, *J* = 1.8 Hz, H-26), 3.93 (3H, *s*, 6′-OCH_3_), 3.66 (3H, *s*, 1-OCH_3_), 2.30 (2H, *t*, *J* = 1.4 Hz, H-2), 1.18–1.23 (46H, *m*, methylene chain). ^13^C-NMR (CDCl_3_) δ_C_ 174.5 (C-1), 167.5 (C-1′), 148.0 (C-6′), 147.9 (C-7′), 144.8 (C-3′), 127.2 (C-4′), 123.2 (C-9′), 118.8 (C-8′), 115.8 (C-2′), 109.4 (C-5′), 64.8(C-26), 56.1 (6′-OCH_3_), 51.6 (1-OCH_3_), 34.3 (C-2), 34.1–24.9 (methylene chain). ESI-MS (rel.int.) *m*/*z* = 625 (100, [M + Na]^+^). ESI-MS *m*/*z* = 601, [M − H]^−^. HR-ESI-MS *m*/*z* = 603.5395, [M + H]^+^ (calculated for C_37_H_63_O_6_, 603.5363).

### 3.4. Cytotoxicity Assay

The human cervix carcinoma cell line KB-3-1 was used in the cytotoxicity assay as previously described [29]. The cell line was cultivated as a monolayer in DMEM (Dulbecco’s modified Eagle medium) with glucose (4.5 g/L), l-glutamine, sodium pyruvate and phenol red, supplemented with 10% fetal bovine serum (FBS) and were maintained at 5.3% CO_2_ and 37 °C in humidified air. The cells at 70% confluence were detached with trypsin-ethylenediamine tetraacetic acid solution (0.05%; 0.02% in DPBS) and placed in sterile 96-well plates in a density of 10,000 cells in 100 μL medium per well. The dilution series of the compounds was prepared from stock solutions in DMSO of concentrations of 100 mM, 50 mM or 25 mM and the stock solutions were diluted with culture medium (10% FBS) down to pM concentrations. The dilution prepared from stock solution was added to the wells and each concentration was tested in at least six replicates. The control contained the same concentration of DMSO as the first dilution. After incubation for 72 h at 37 °C and 5.3% CO_2_-humidified air, 30 μL of an aqueous resazurin solution (175 μM) was added to each well. The cells were incubated at the same conditions for 5 h. Subsequently, the fluorescence was recorded at a wavelength of 588 nm. The IC_50_ values were calculated as a sigmoidal dose response curve using Graphpad Prism 4.03 (Graphpad software Inc., San Diego, CA, USA).

## 4. Conclusions

Four new natural products along with ten other known compounds were identified from the root of *A. megalacantha*. The two aloesaponarins (aloesaponarin I and II) showed good cytotoxic activity even greater than one of the reference drug (griseofulvin) against KB-3-1 human cervical cancer cell line, with the highest activity observed for aloesaponarin II.

## Figures and Tables

**Table 1:** ^1^H (500 MHz) and ^13^C (125 MHz) NMR data of compound **1**, **2** (acetone-*d*_6_) and **3** (CDCl_3_)

Position	1		2		3	
	δ_H_ (m, *J* in Hz)	δ_C_	δ_H_ (m, *J* in Hz)	δ_C_	δ_H_ (m, *J* in Hz)	δ_C_
1		154.8		142.3		162.8
1a				125.3		117.0
2		132.0		130.8	6.76 (d, 1.5)	116.9
3		133.6		159.0		150.8
4	7.48 (br.s)	125.6	7.75 (s)	112.8	6.99 (d, 1.5)	115.0
4a		138.6		138.3		144.1
5	7.40 (dd, 7.8, 1.4)	121.0	8.19 (dd, 7.7, 1.4)	127.2	6.77 (dd, 8.0, 1.1)	119.3
5a				133.5		143.6
6	7.44 (t, 7.8)	128.5	7.86 (td, 7.5, 1.4)	134,4	7.43 (t, 8.2)	136.8
7	6.97 (dd, 7.6, 1.2)	106.7	7.91 (td, 7.5, 1.5)	135.5	6.95 (dd, 8.5, 1.1)	117.8
8		157.0	8.24 (dd, 7.7, 1.5)	128.0		162.4
8a		118.7		136.2		116.2
9				184.0		193.1
10				183.5		75.6
1′						162.7
1′a						115.8
2′					7.02 (d, 1.6)	124.5
3′						149.6
4′					7.60 (d, 1.5)	121.5
4′a						133.3
5′					7.89 (d, 8.0)	119.6
5′a						132.9
6′					8.85 (d, 8.0)	132.7
7′						141.7
8′						158.8
8′a						115.8
9′						192.5
10′						182.0
2-*C*O		207.5		168.3		
2/3-*CH*_2_-OH	4.55 (s)	70.5			4. 63 (s)	64.5
2-COO*Me*			3.95 (s)	52.9		
1/3/3′-Me	2.30 (d, 1.0)	19.0	2.71 (s)	20.0	2.43 (s)	22.4
1-OMe	3.73 (s)	64.1				
8/10-OMe	3.99 (s)	56.3			2.87 (s)	50.7
1-OH					12.39 (s)	
1′-OH					11.76 (s)	
8-OH					12.36 (s)	
8′-OH					11.76 (s)	

## Supplementary Materials

Supplementary materials are available online.The MS and NMR data of compounds **1**–**4** are available online.
