# Supplementary material for: Cytotoxic Compounds from Aloe megalacantha
Source: Molecules. 2017 Jul 7;22(7):1136. doi: 10.3390/molecules22071136 (PMC6152336; doi:10.3390/molecules22071136)
Supplement: Supplementary file 1 [file molecules-22-01136-s001.pdf]

Supporting Information

Cytotoxic Compounds from *Aloe megalacantha*

Negera Abdissa, Sebastian Gohlke, Marcel Frese, and Norbert Sewald

Table of contents

Contents **Page**

Spectroscopic data for 1,8-dimethoxynepodinol (1)

Figure S1: ^{1}H NMR spectrum of 1,8-dimethoxynepodinol (**1**) S2
Figure S2: ^{13}C NMR spectrum of 1,8-dimethoxynepodinol (**1**) S2
Figure S3: COSY spectrum of 1,8-dimethoxynepodinol (**1**) S3
Figure S4: HMQC spectrum of 1,8-dimethoxynepodinol (**1**) S3
Figure S5: HMBC spectrum of 1,8-dimethoxynepodinol (**1**) S4
Figure S6: ESI-MS spectra of 1,8-dimethoxynepodinol (**1**) S4
Figure S7: HRESIMS spectra of 1,8-dimethoxynepodinol (**1**) S5

Spectroscopic data for aloesaponarin III (2)

Figure S8: ^{1}H NMR spectrum of aloesaponarin III (**2**) S5
Figure S9: ^{13}C NMR spectrum of aloesaponarin III (**2**) S6
Figure S10: COSY spectrum of aloesaponarin III (**2**) S6
Figure S11: HMQC spectrum of aloesaponarin III (**2**) S7
Figure S12: HMBC spectrum of aloesaponarin III (**2**) S7
Figure S13: HRESIMS spectra of aloesaponarin III (**2**) S8

Spectroscopic data for 10-*O*-methylchrysalodin (3)

Figure S14: ^{1}H NMR spectrum of 10-*O*-methylchrysalodin (**3**) S8
Figure S15: ^{13}C NMR spectrum of 10-*O*-methylchrysalodin (**3**) S9
Figure S16: COSY spectrum of 10-*O*-methylchrysalodin (**3**) S9
Figure S17: NOESY spectrum of 10-*O*-methylchrysalodin (**3**) S10
Figure S18: HMQC spectrum of 10-*O*-methylchrysalodin (**3**) S10
Figure S19: HMBC spectrum of 10-*O*-methylchrysalodin (**3**) S11
Figure S20: HRESIMS spectra of 10-*O*-methylchrysalodin (**3**) S11
Figure S21: CD spectra of 10-*O*-methylchrysalodin (**3**) S11

Spectroscopic data for methyl 26-*O*-feruloyl-oxyhexacosanoate (4)

Figure S22: ^{1}H NMR spectrum of methyl 26-*O*-feruloyl-oxyhexacosanoate (**4**) S12
Figure S23: ^{13}C NMR spectrum of methyl 26-*O*-feruloyl-oxyhexacosanoate (**4**) S12
Figure S24: COSY spectrum of methyl 26-*O*-feruloyl-oxyhexacosanoate (**4**) S13
Figure S25: HMQC spectrum of methyl 26-*O*-feruloyl-oxyhexacosanoate (**4**) S13
Figure S26: HMBC spectrum of methyl 26-*O*-feruloyl-oxyhexacosanoate (**4**) S14
Figure S27: ESI-MS spectra of methyl 26-*O*-feruloyl-oxyhexacosanoate (**4**) S14

Spectroscopic data for 1,8-dimethoxynepodinol (1)

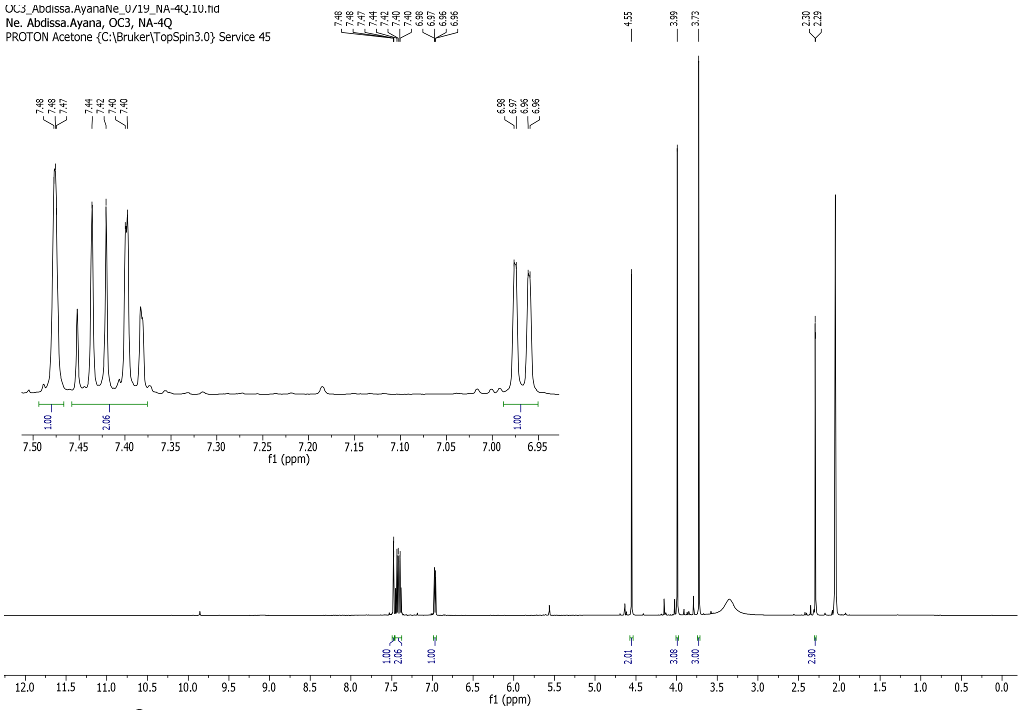

Figure S1. The ^{1}H NMR spectrum of 1,8-dimethoxynepodinol (**1**) observed at 500 MHz in acetone-d_6 at 25 °C. Assignments are given in Table 1.

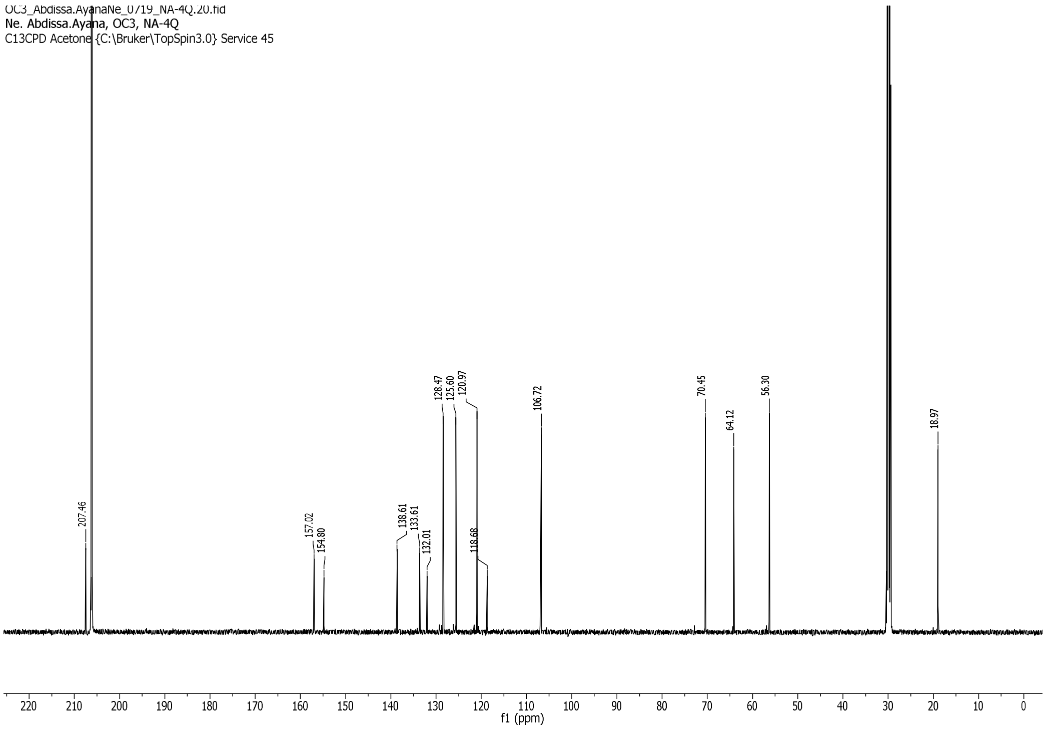

Figure S2. The ^{13}C NMR spectrum of 1,8-dimethoxynepodinol (**1**) observed at 125 MHz for acetone-d_6 solution at 25 °C. Assignments are given in Table 1.

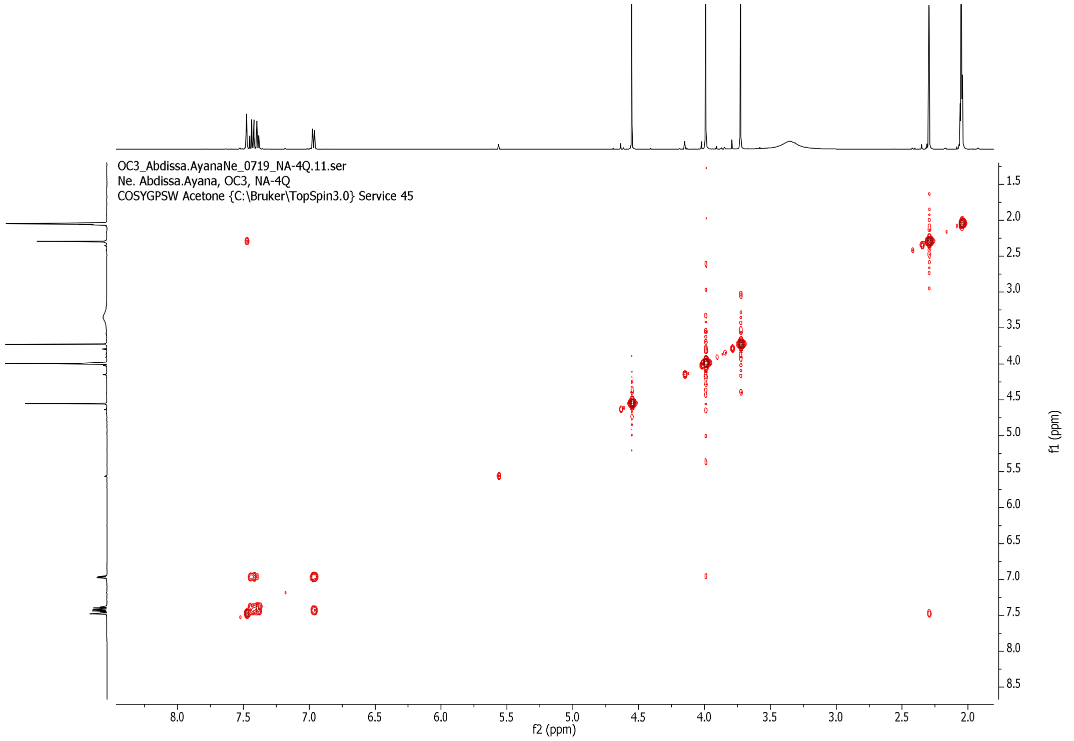

Figure S3. The COSY spectrum of 1,8-dimethoxynepodinol (**1**) observed at 500 MHz in acetone-d_6 solution at 25 °C.

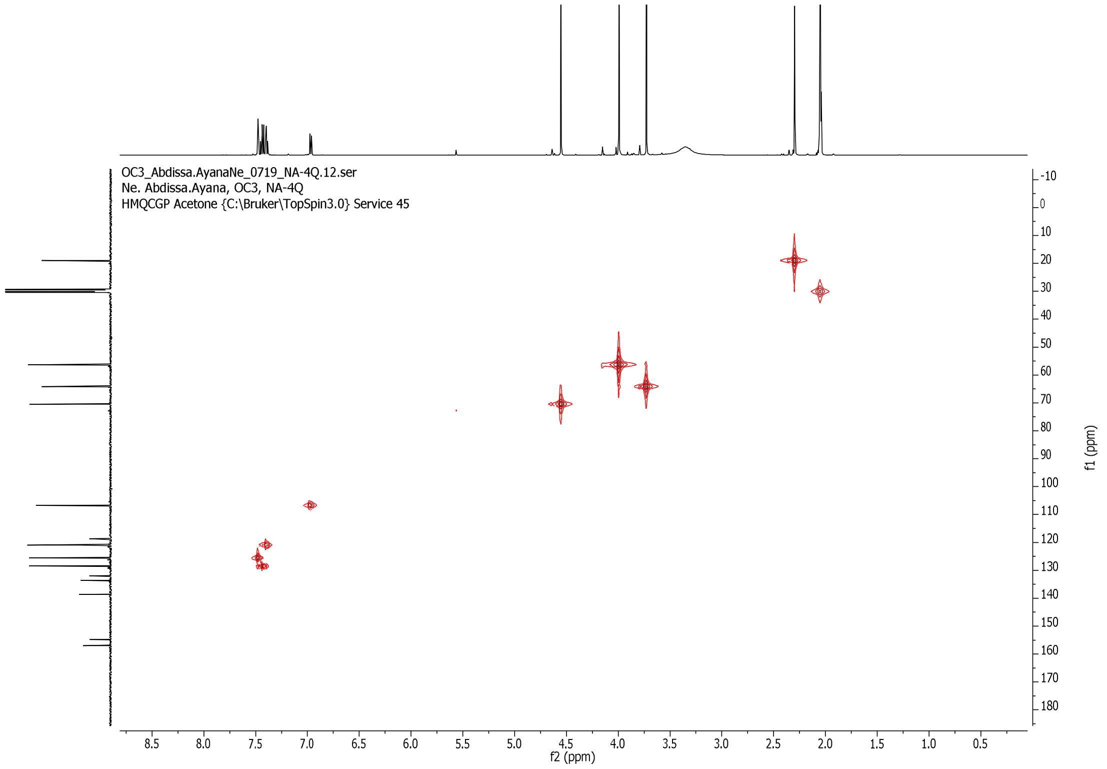

Figure S4. The HSQC spectrum of 1,8-dimethoxynepodinol (**1**) observed at 500 and 125 MHz for acetone-d_6 solution at 25 °C.

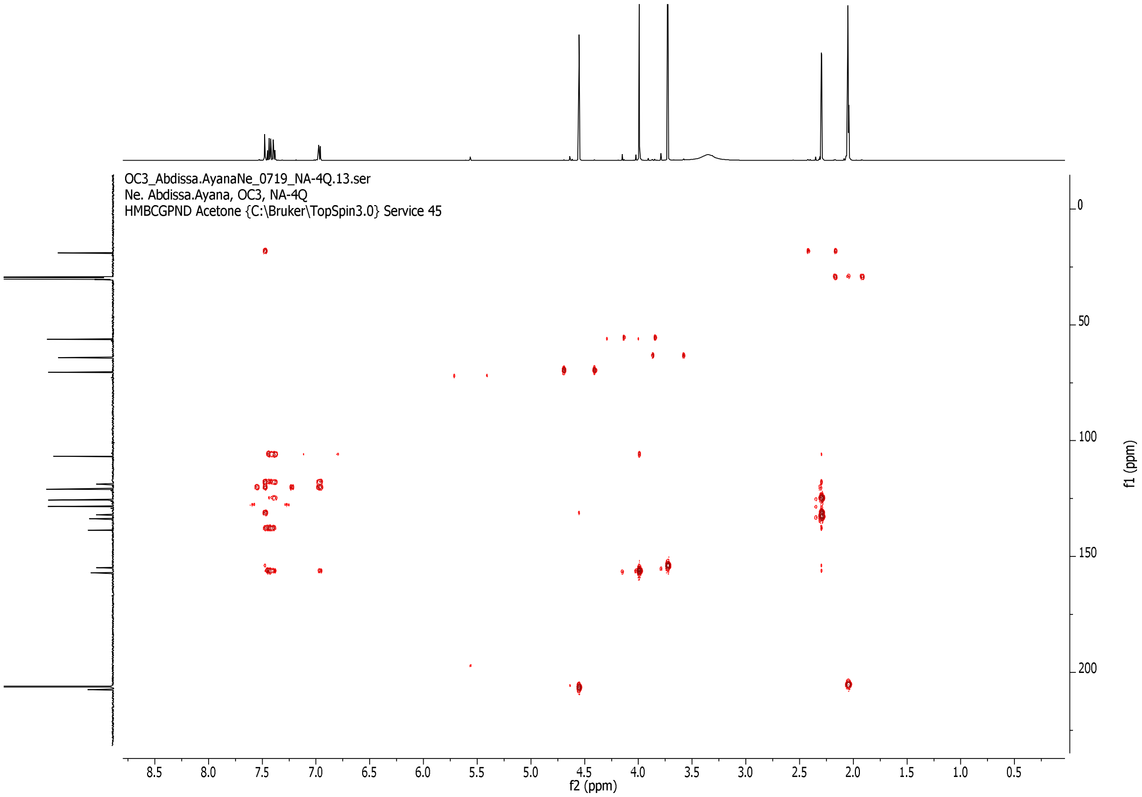

Figure S 5. The HMBC spectrum of 1,8-dimethoxynepodinol (**1**) observed at 500 and 125 MHz for acetone-d_6 solution at 25 °C.

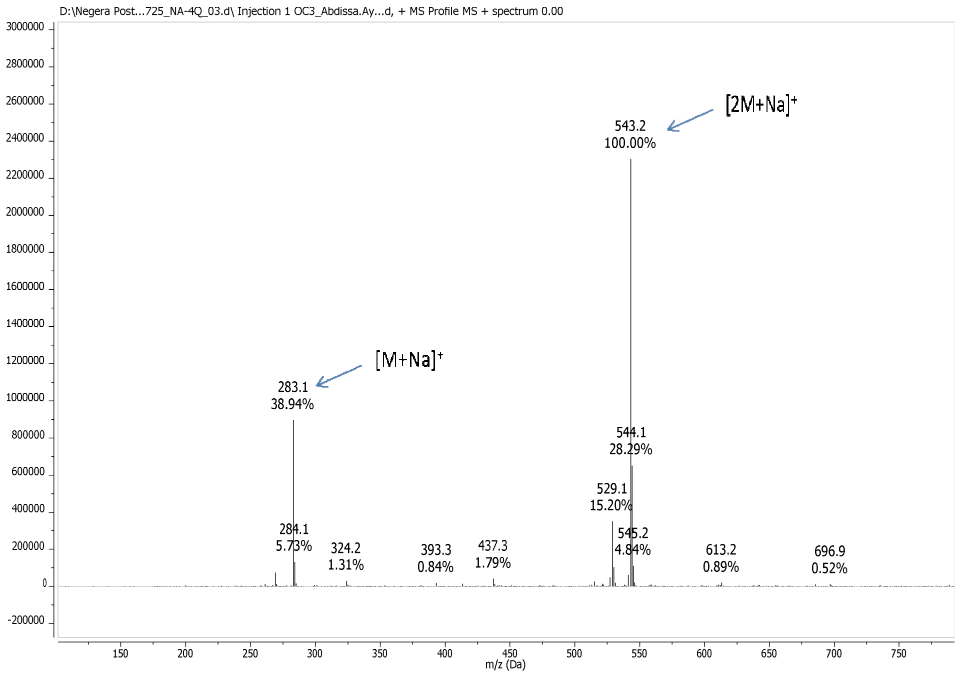

Figure S 6. The ESI-MS of 1,8-dimethoxynepodinol (**1**).

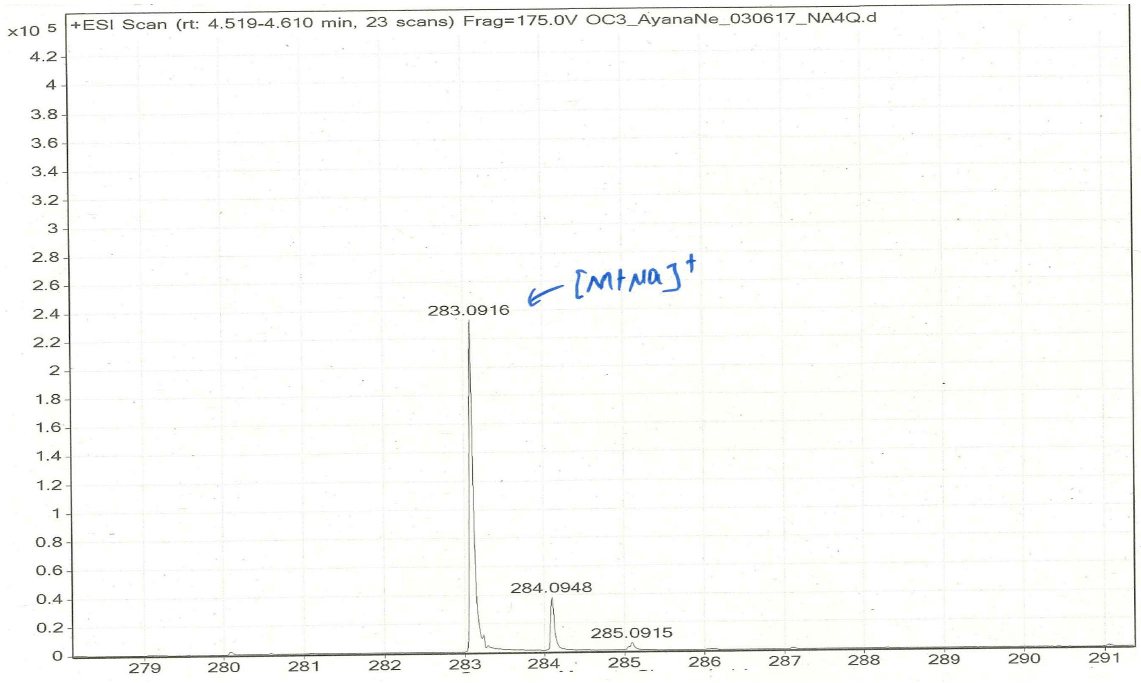

Figure S7. The HRESIMS of 1,8-dimethoxynepodinol (**1**).

Spectroscopic data for aloesaponarin III B (2)

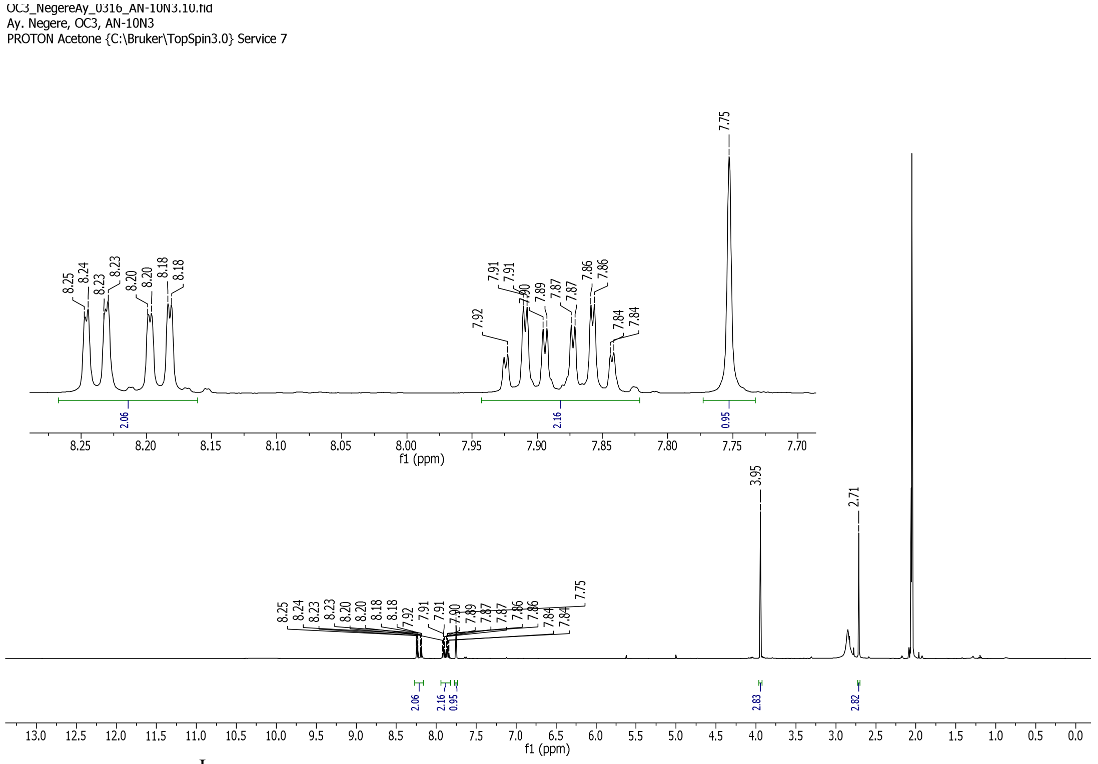

Figure S8. The ^{1}H NMR of aloesaponarin III (**2**) observed at 500 MHz in acetone-d_6 solution at 25 °C. Assignments are given in Table 1.

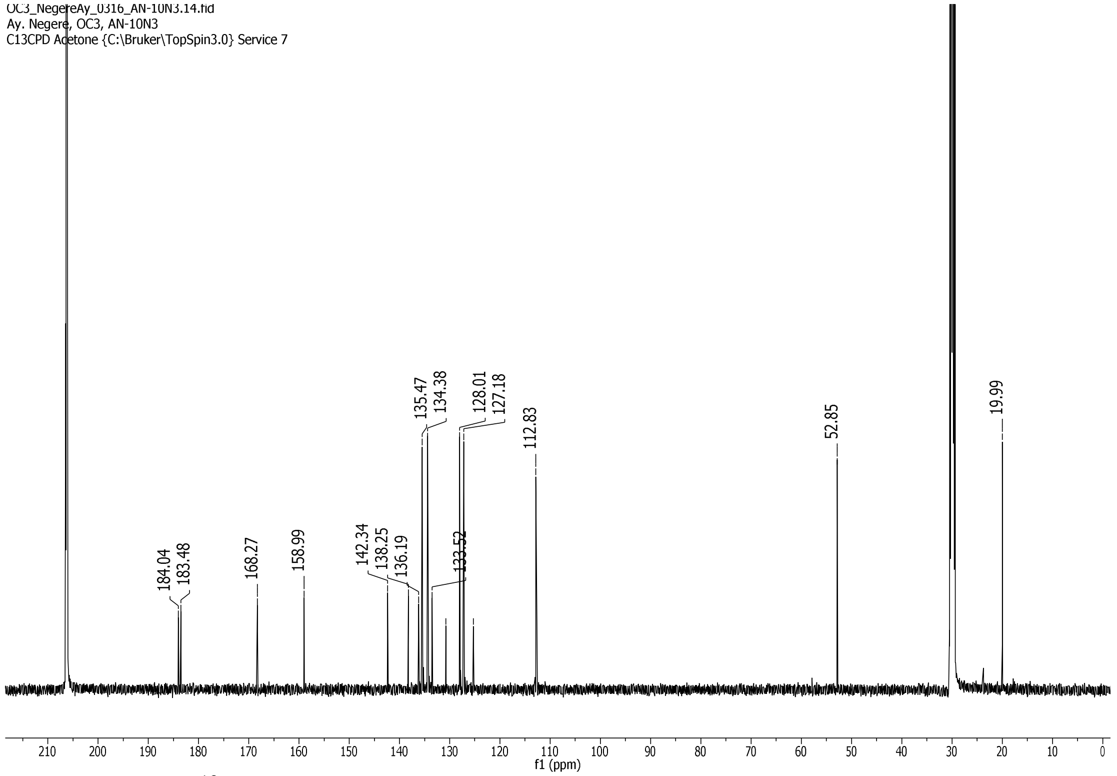

Figure S9. The ^{13}C NMR spectrum of aloesaponarin III (**2**) observed at 125 MHz for acetone-d_6 solution at 25 °C. Assignments are given in Table 1.

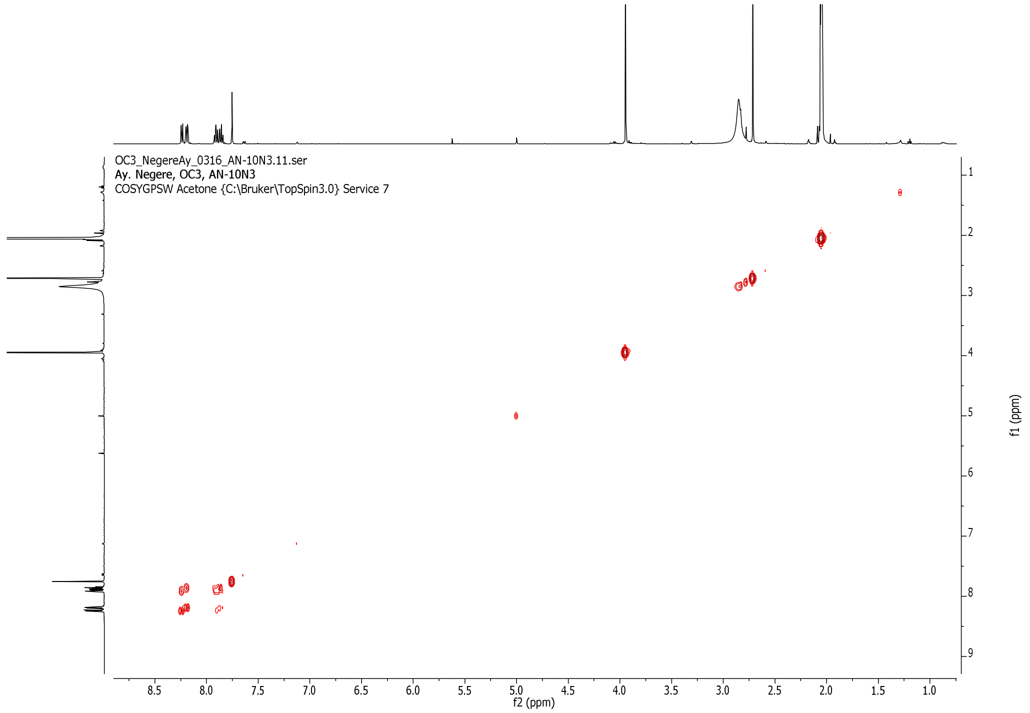

Figure S10. The COSY spectrum of aloesaponarin III (**2**) observed at 500 MHz in acetone-d_6 solution at 25 °C.

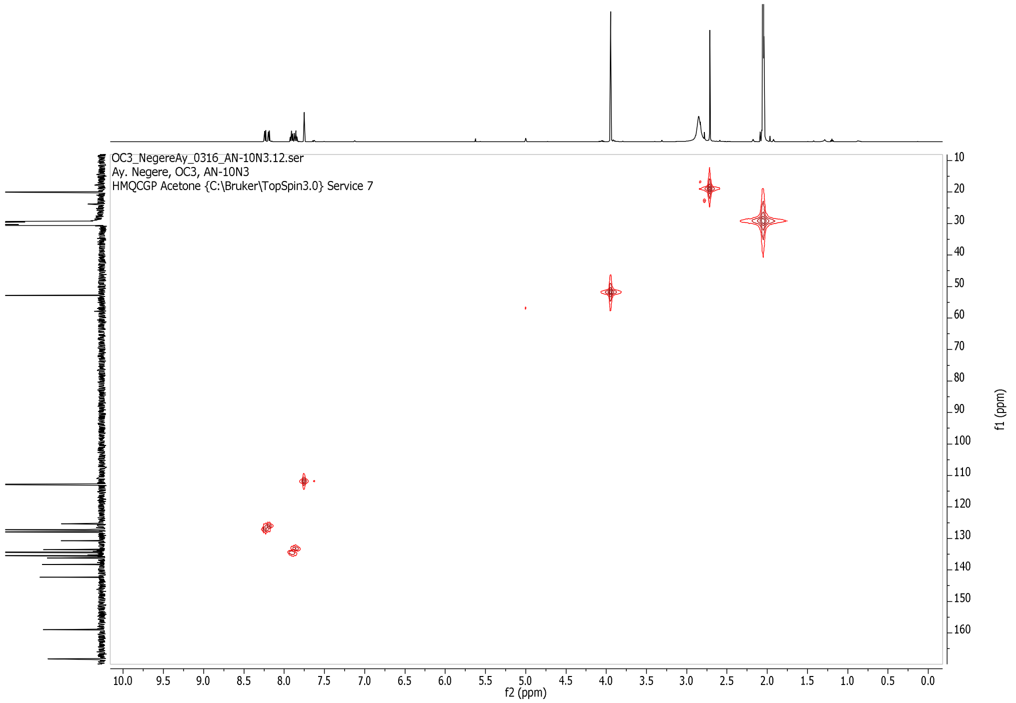

Figure S11. The HSQC spectrum of aloesaponarin III (**2**) observed at 500 and 125 MHz for acetone-d_6 solution at 25 °C.

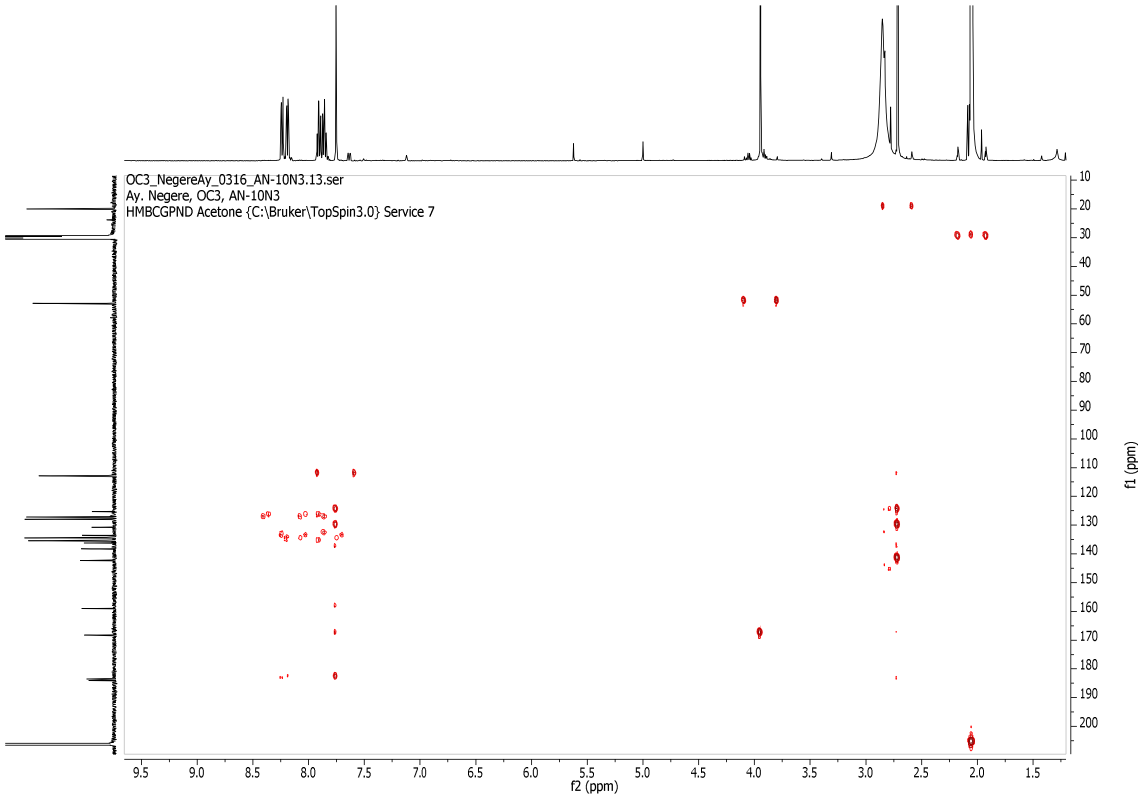

Figure S12. The HMBC spectrum of aloesaponarin III (**2**) observed at 500 and 125 MHz for acetone-d_6 solution at 25 °C.

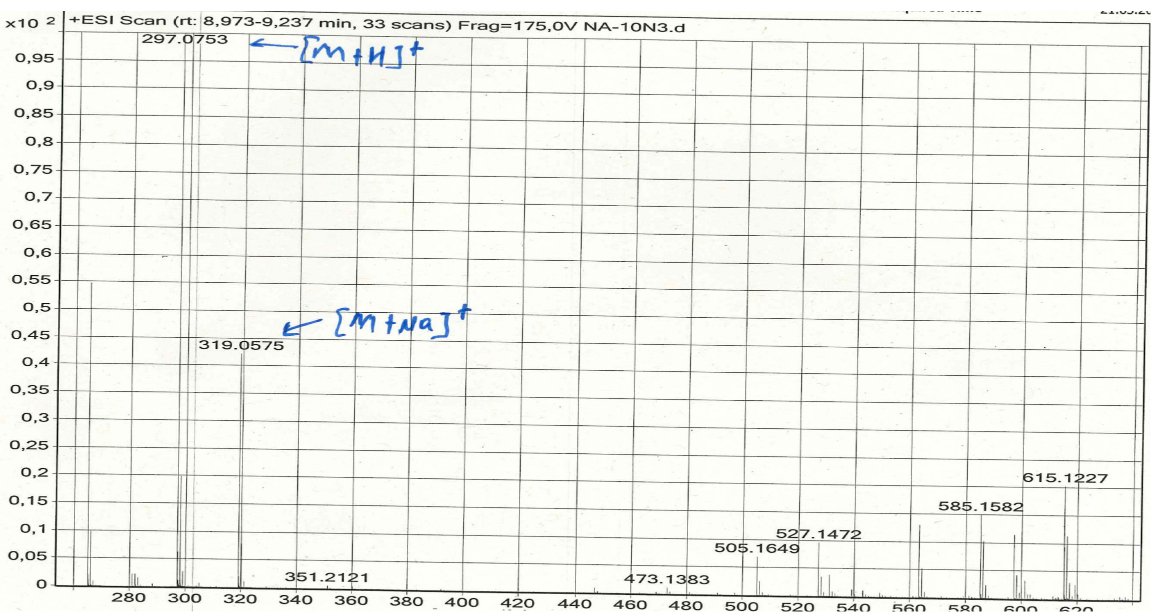

Figure S13. The HRESIMS of aloesaponarin III (**2**)

Spectroscopic data for 10-*O*-methylchrysalodin (3)

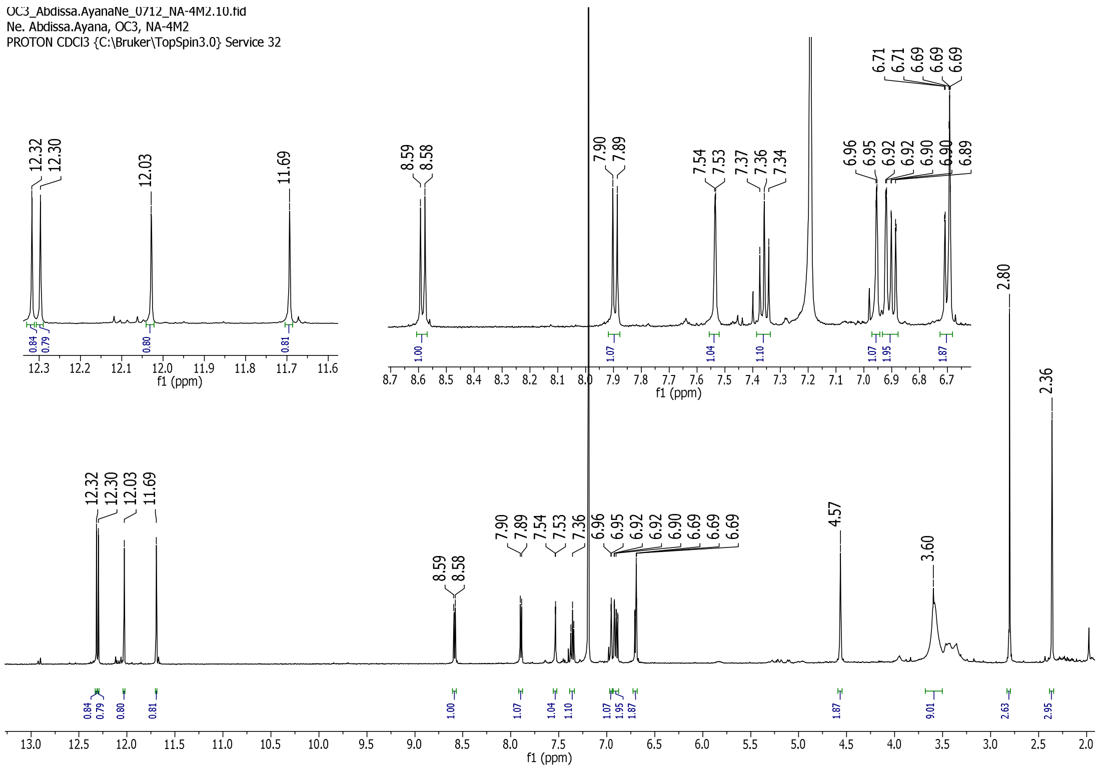

Figure S14. The ^{1}H NMR of 10-*O*-methylchrysalodin (**3**) observed at 500 MHz for $CDCl_3$ solution at 25 °C. Assignment is given in Table 1.

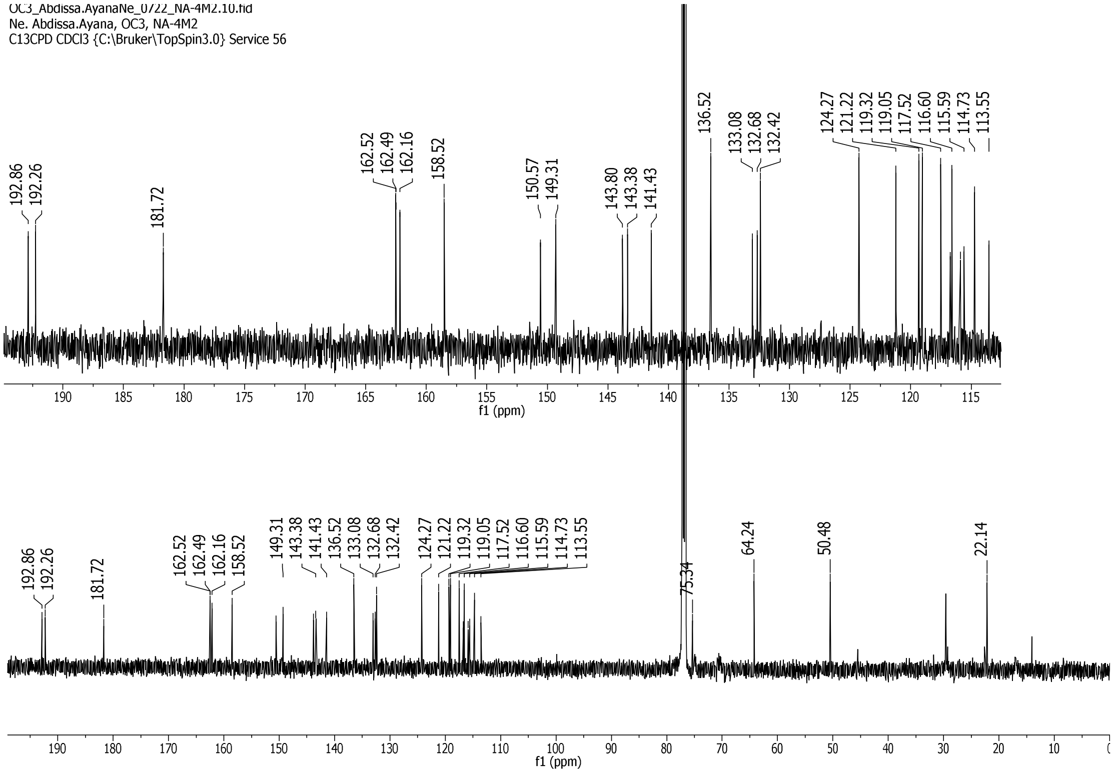

Figure S15. The ^{13}C NMR spectrum of 10-*O*-methylchrysalodin (**3**) observed at 125 MHz for $CDCl_3$ solution at 25 °C. Assignment is given in Table 1.

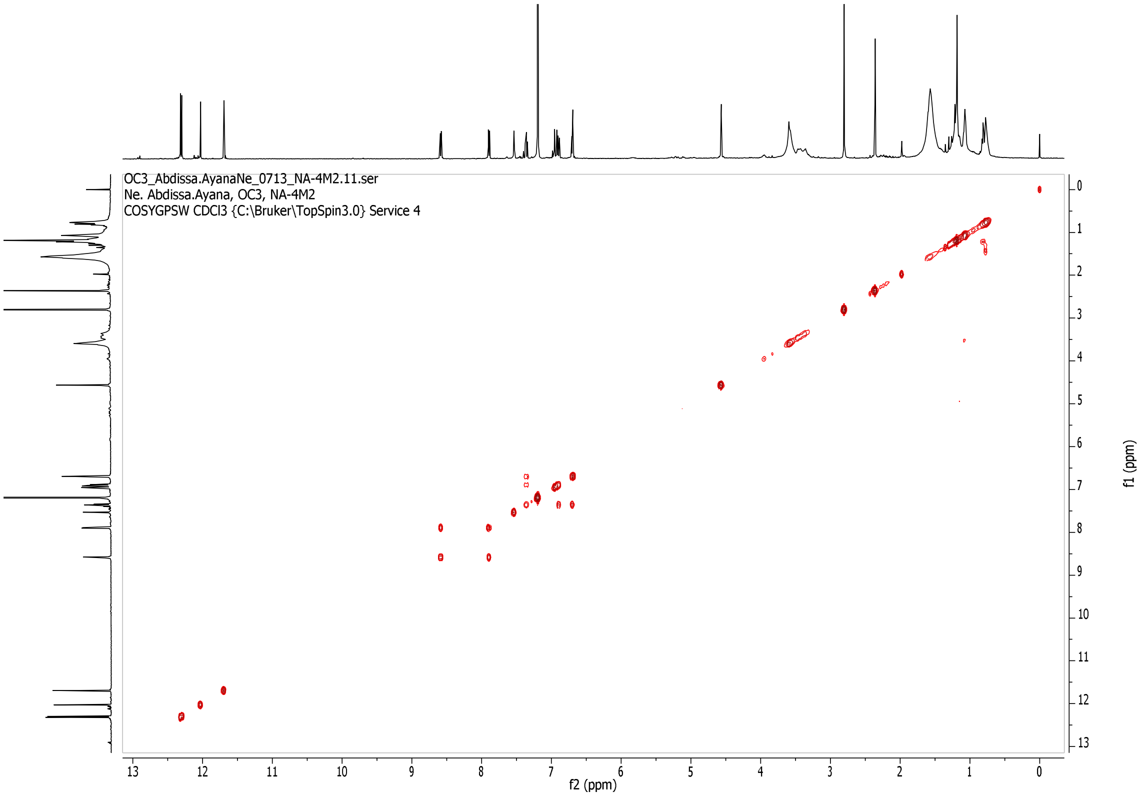

Figure S16. The COSY spectrum of 10-*O*-methylchrysalodin (**3**) observed at 500 MHz for $CDCl_3$ solution at 25 °C.

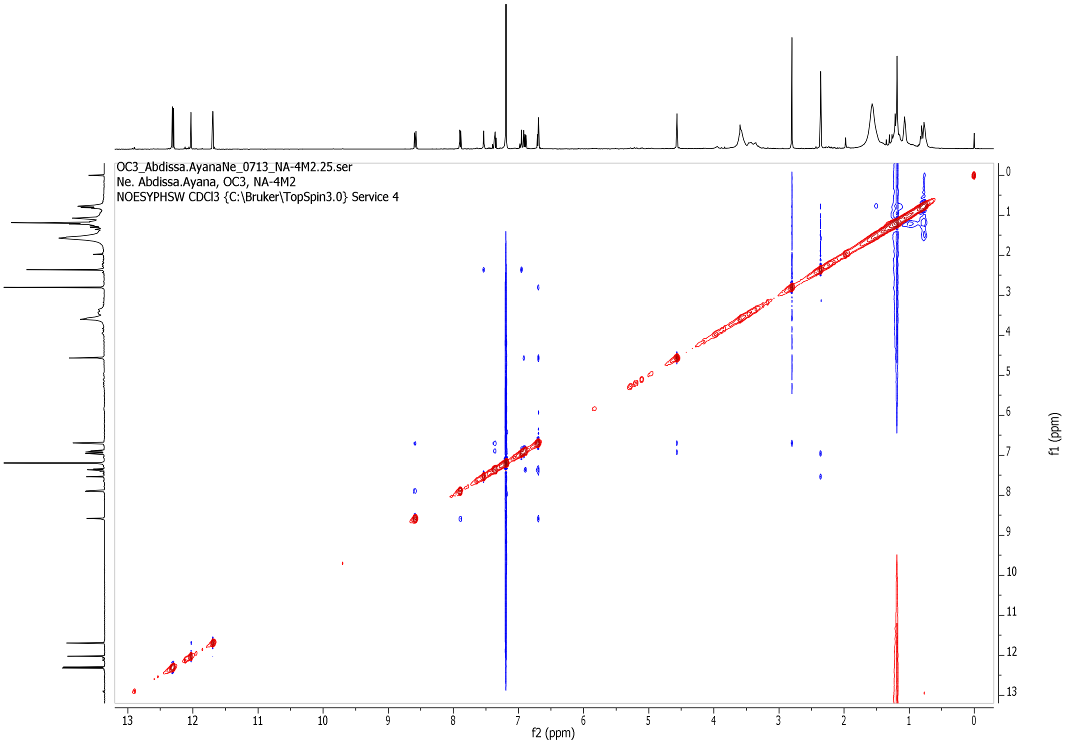

Figure S17. The NOESY spectrum of 10-*O*-methylchrysalodin (**3**) observed at 500 MHz for $CDCl_3$ solution at 25 °C.

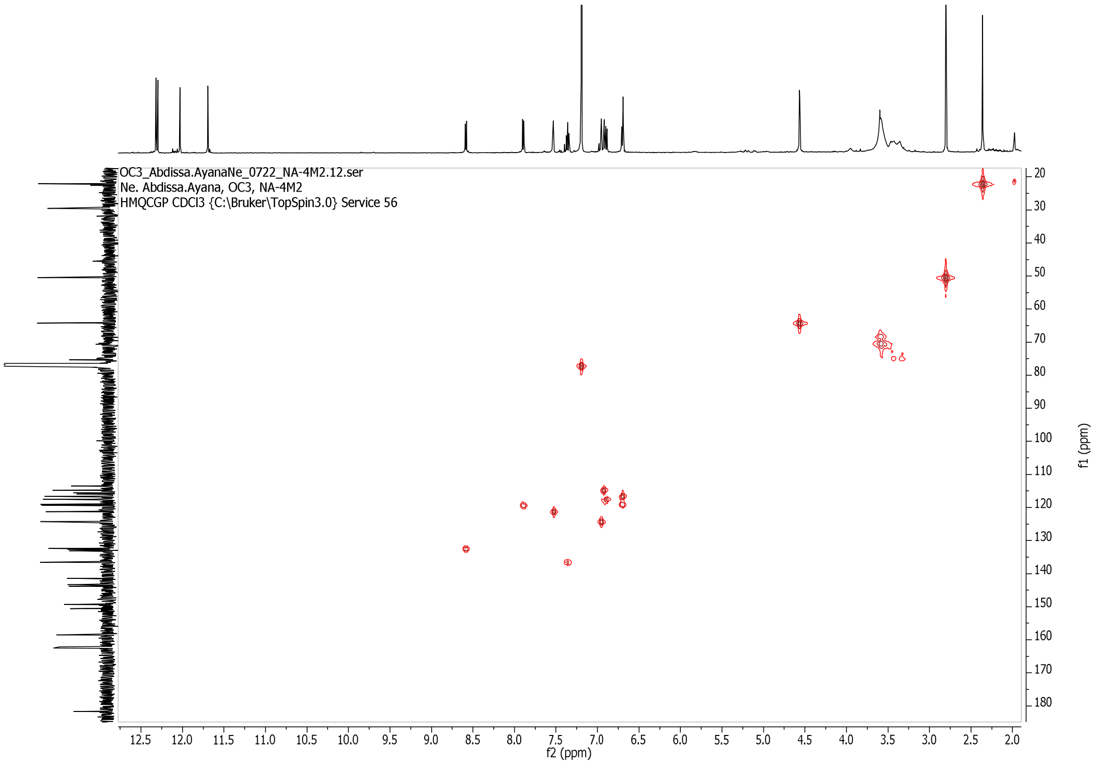

Figure S18. The HSQC spectrum of 10-*O*-methylchrysalodin (**3**) observed at 500 and 125 MHz for $CDCl_3$ solution at 25 °C.

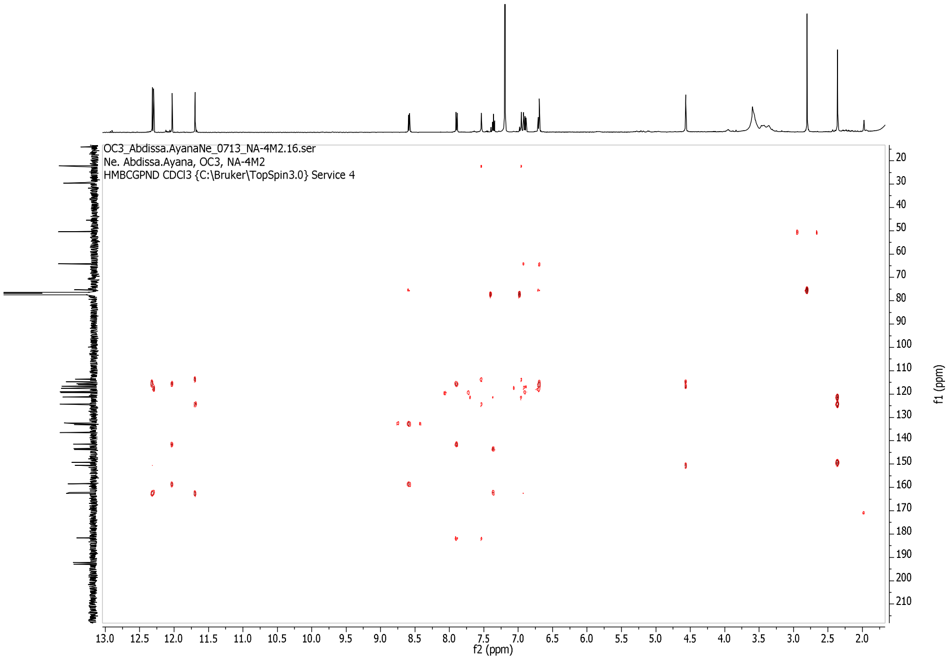

Figure S19. The HMBC spectrum of 10-*O*-methylchrysalodin (**3**) observed at 500 and 125 MHz for $CDCl_3$ solution at 25 °C.

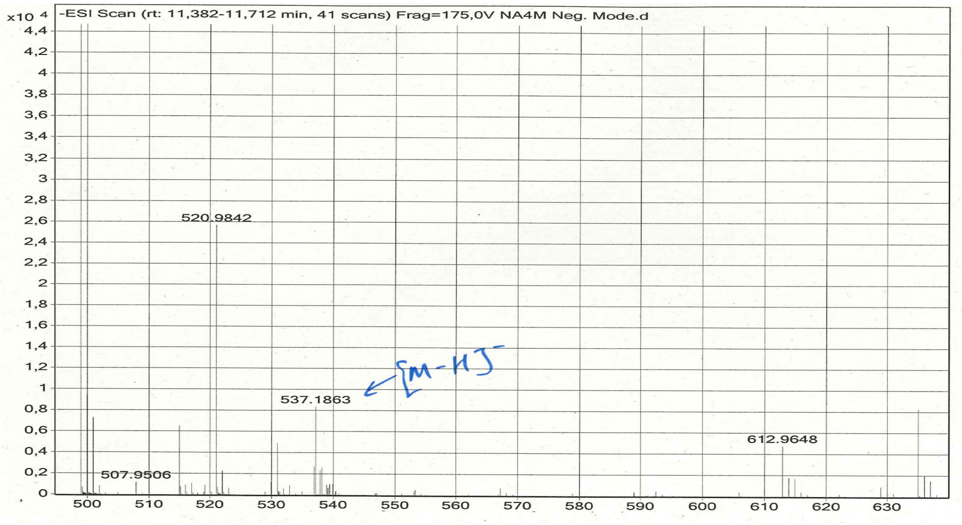

Figure S20. The HRESIMS of 10-*O*-methylchrysalodin (**3**).

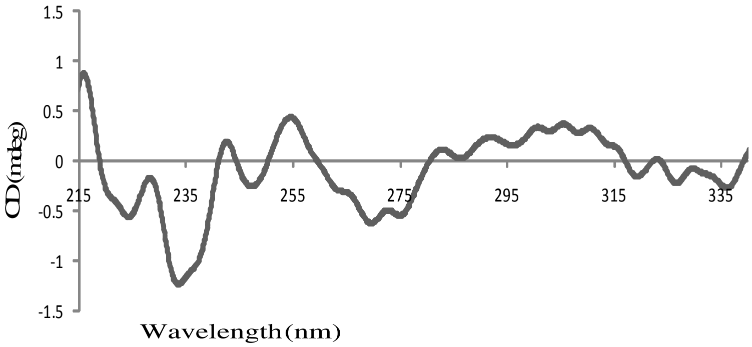

Figure21. The CD spectrum of 10-*O*-methylchrysalodin (**3**) in acetonitril

Spectroscopic data for methyl 26-*O*-feruloyl-oxyhexacosanoate (4)

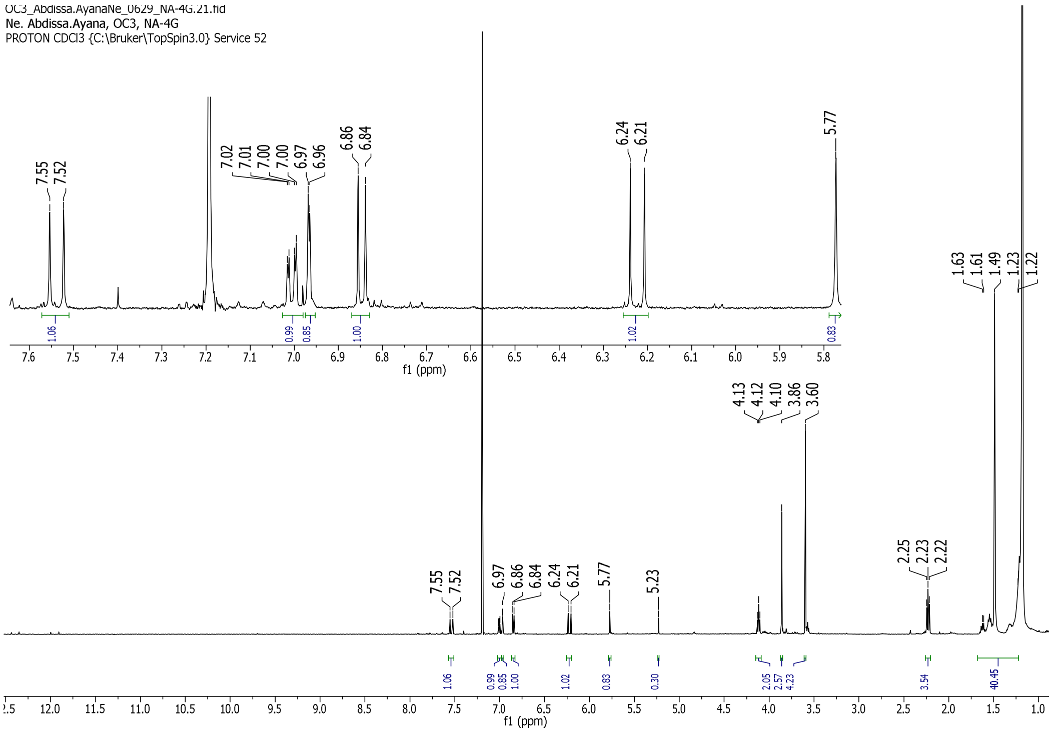

Figure S22. The [I]H NMR of methyl 26-*O*-feruloyl-oxyhexacosanoate (**4**) observed at 500 MHz for $CDCl_3$ solution at 25 °C. Assignment is given in section 3.3.

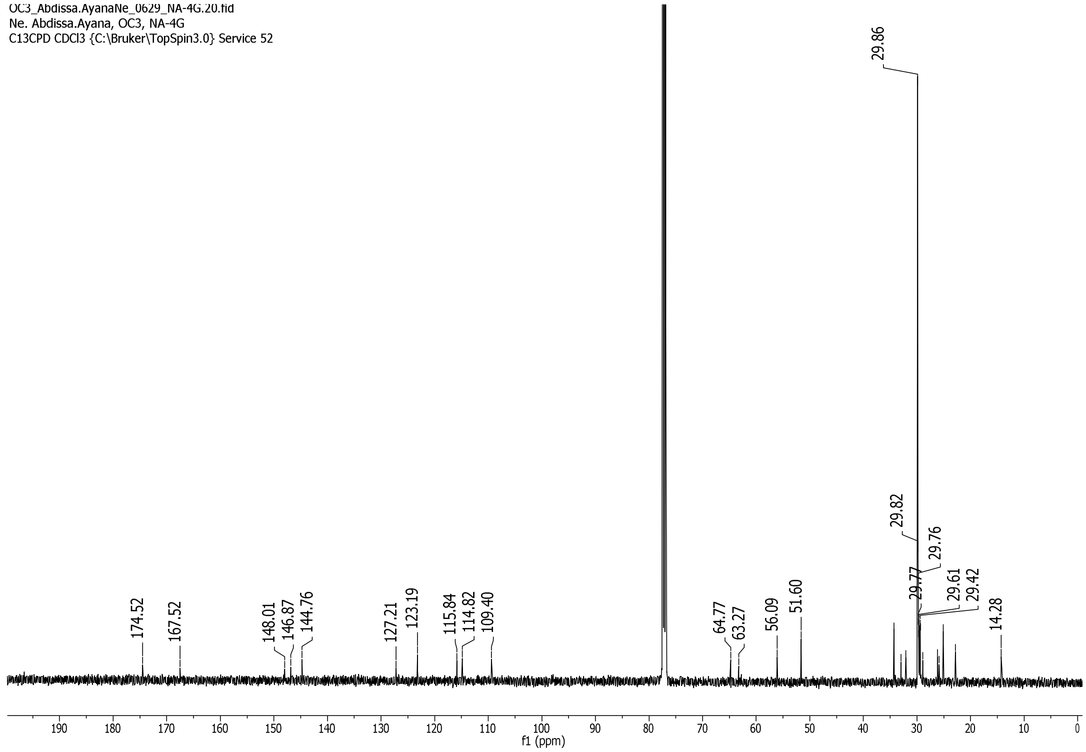

Figure S23. The ^{13}C NMR spectrum methyl 26-*O*-feruloyl-oxyhexacosanoate (**4**) observed at 125 MHz for $CDCl_3$ solution at 25 °C. Assignment is given in section 3.3.

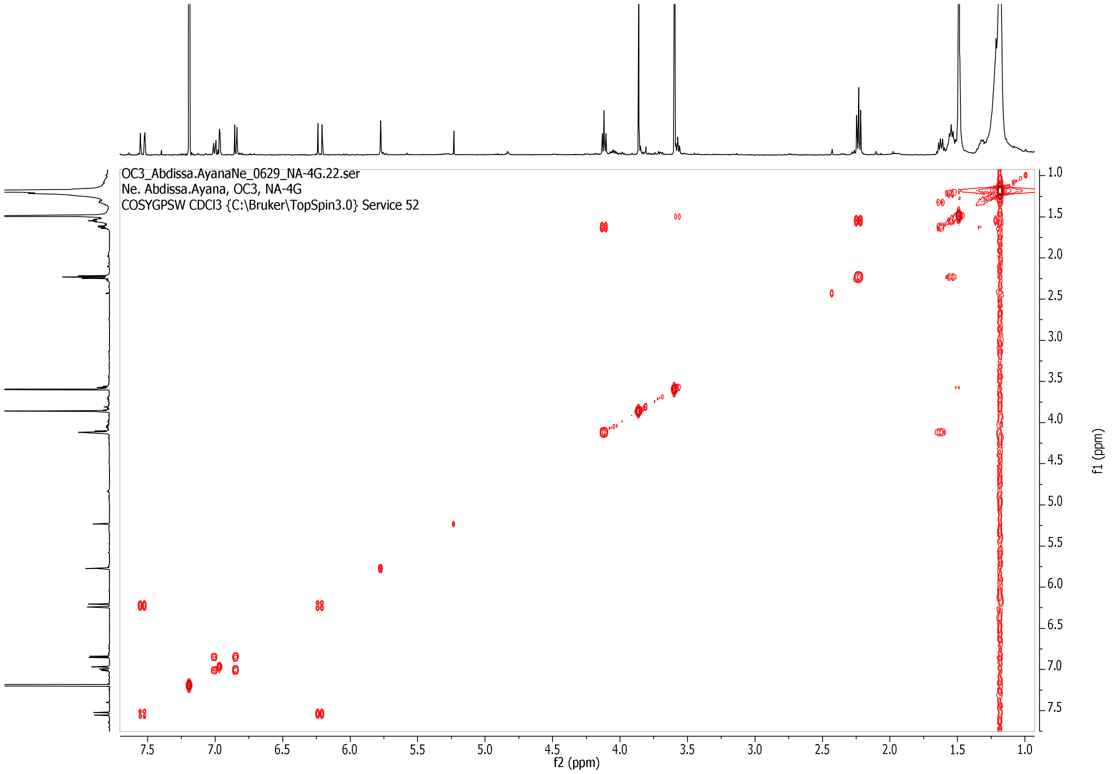

Figure S24. The COSY spectrum of methyl 26-*O*-feruloyl-oxyhexacosanoate (**4**) observed at 500 MHz for $CDCl_3$ solution at 25 °C.

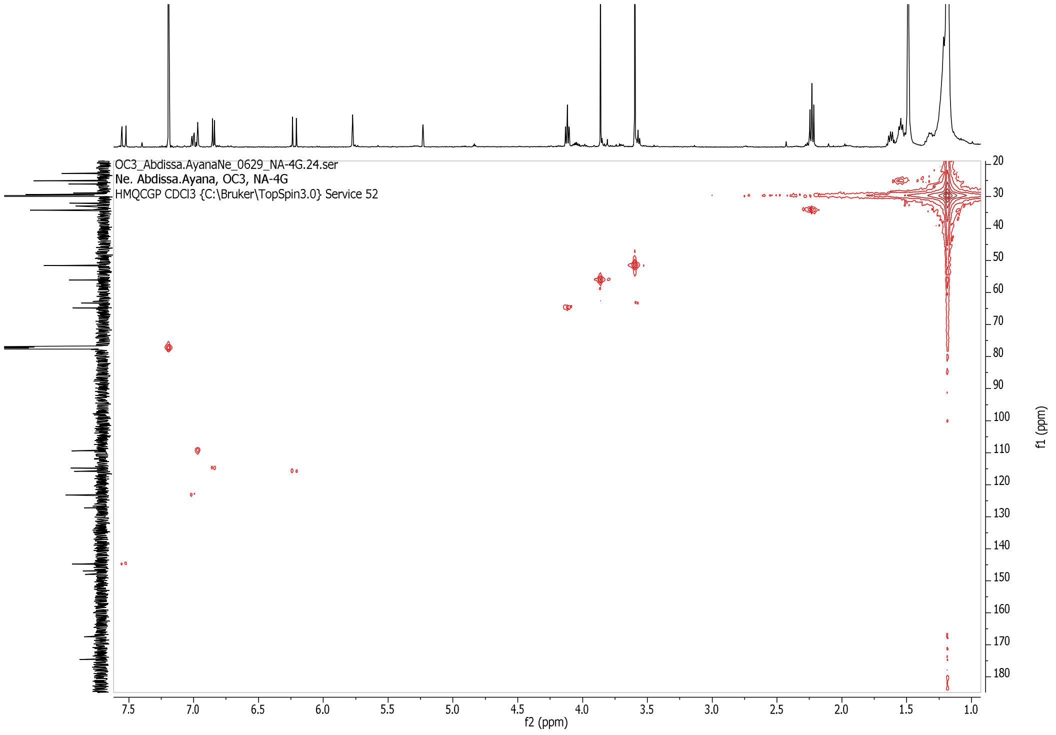

Figure S25. The HSQC spectrum of methyl 26-*O*-feruloyl-oxyhexacosanoate (**4**) observed at 500 and 125 MHz for $CDCl_3$ solution at 25 °C.

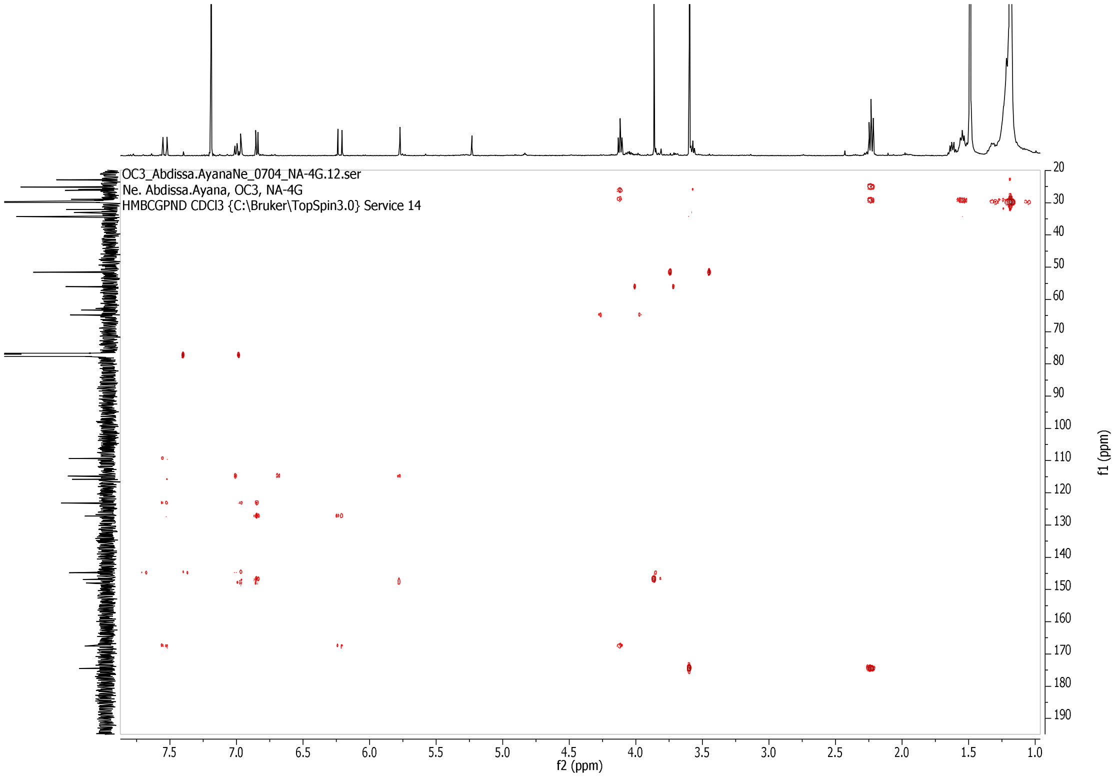

Figure S26. The HMBC spectrum of methyl 26-*O*-feruloyl-oxyhexacosanoate (**4**) observed at 500 and 125 MHz for $CDCl_3$ solution at 25 °C.

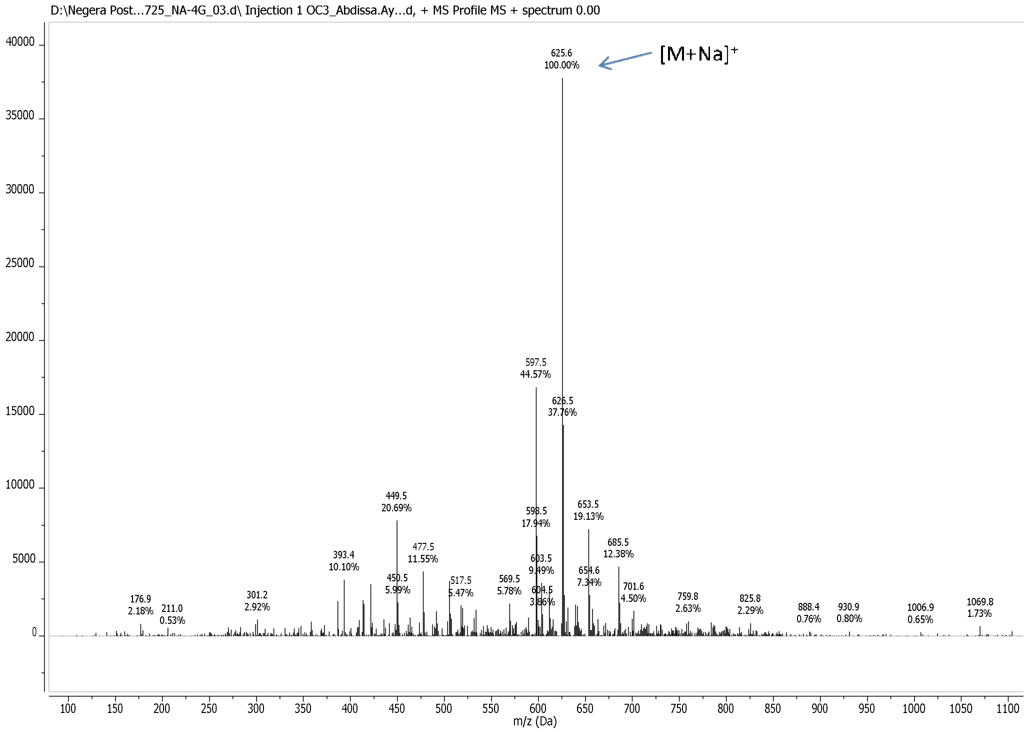

Figure S27. The ESI-MS of methyl 26-*O*-feruloyl-oxyhexacosanoate (**4**)
